# Increased risk of anxiety and coping strategies in patients with selected genodermatoses with cornification disruption

**DOI:** 10.1038/s41598-025-98535-6

**Published:** 2025-04-23

**Authors:** Magdalena Fryze, Radoslaw Mlak, Aleksandra Kulbaka, Katarzyna Wertheim-Tysarowska, Dariusz Matosiuk, Aldona Pietrzak

**Affiliations:** 1 Department of Psychology, Psychosocial Aspects of Medicine, Medical University of Lublin, Lublin, Poland; 2 Department of Laboratory Diagnostics, Medical University of Lublin, Lublin, Poland; 3 Imperial College London, London, UK; 4 Department of Medical Genetics, Institute of Mother and Child, Warsaw, Poland; 5 Department of Synthesis and Chemical Technology of Pharmaceutical Substances, Faculty of Pharmacy, Medical University of Lublin, Lublin, Poland; 6 Department of Dermatology, Venereology and Paediatric Dermatology, Medical University of Lublin, Staszica 11, Lublin, 20-080 Poland

**Keywords:** Genodermatoses, Ichthyosis, Palmoplantar keratoderma, Anxiety, coping strategies, Psychology, Diseases, Medical research

## Abstract

People with genodermatoses face physical pain, social discrimination, and daily life challenges, all of which have an impact on their emotional and psychological well-being. The assessment of anxiety and the development of coping strategies are crucial. This study aimed to compare state and trait anxiety between healthy adults (*n* = 30) and patients with Mendelian Disorders of cornification (MeDOC) (*n* = 29). Using the State and Trait Anxiety Inventory and Coping in Stressful Situations Questionnaire, we compared anxiety levels and coping strategies between patients with MeDOC and healthy controls. Given the rarity of MeDOC, the study group is small, but the findings are highly relevant and can significantly improve patients’ well-being. Average or high levels of trait anxiety were significantly more common in the study group compared to the control group (25 vs. 18 cases; 86.2% vs. 60%, respectively; *p* = 0.0488). It was estimated that the risk of average or a high level of trait anxiety was over 4 times higher in the study group than in the control group (OR = 4.2, 95% CI 1.1–15; *p* = 0.0293). High-level emotion-oriented coping was significantly more frequent in the study group compared to the control group (8 vs. 1 case; 27.6% vs. 3.3%; *p* = 0.0259), with the risk being 11 times higher in the study group (OR = 11, 95% CI 1.3–95.2; *p* = 0.0288). No significant correlation was found between demographic, social, educational, clinical factors, and anxiety levels or avoidant-distracted coping. Patients with MeDOC have an increased risk of experiencing anxiety. Understanding the emotions and behaviors of patients with this disease is essential for clinical specialists to guide their coping strategies effectively.

## Introduction

Genodermatoses with cornification disruption manifest either as localized (e.g., Palmoplantar Keratoderma - PPK) or generalized (e.g., ichthyoses) hyperkeratosis and are often referred to as Mendelian Disorders of Cornification (MeDOC). Ichthyoses are a group of disorders of keratinization characterized by dry, rough skin and prominent scaling^1^. These skin conditions often involve varying degrees of pruritus, erythema and impaired sweating^2^. Visible skin lesions on the face and arms significantly affect patients’ well-being, leading to lowered self-esteem, feelings of shame, social isolation, sadness, anxiety and irritability^3–5^. Sufferers have to cope with the physical discomfort and psychological effects of unrealistic appearance standards, often leading to the coexistence of psychological disorders such as depression and anxiety^6–8^. Negative social reactions such as stares, unwanted comments or intimidation can cause embarrassment and shame, hindering relationships and connections. In situations that require exposure of the body, patients often feel humiliation and discomfort. Skin diseases have a negative impact on patients’ work lives, as frequent visits to doctors, complicated care procedures and painful symptoms reduce their ability to work. In addition, the unpredictability of the course of the disease and the variability of symptoms mean that patients have to cope not only with the daily difficulties of caring for their condition but also with the high cost of treatment, which includes regular follow-up visits and specialized procedures. All these factors raise stress levels and reduce patients’ quality of life^9–14^. Understanding these challenges in routine clinical practice plays a crucial role in determining how to appropriately manage patients’ conditions^15^. Coping with stress involves managing demands that are perceived as overwhelming or beyond one’s capabilities^16^. There are three main styles of coping with stress: task-oriented, emotion-oriented, and avoidance. The task-oriented style characterizes individuals who, in stressful situations, focus on action, making efforts to solve the problem. They try to change or transform the situation, concentrating on facts without significant emotional involvement. The emotion-oriented style includes individuals who focus on their feelings and seek ways to reduce tension without thinking about solving the problem. They often worry, blame themselves for failures, and seek support from others. The avoidance style characterizes individuals who try not to think about stressors and who avoid associated emotions. This may take the form of engaging in substitute activities such as watching TV, sleeping, overeating, or seeking social contacts^16–18^. Patients with ichthyoses may employ different strategies to cope with increased stress levels, i.e., they may focus on their own emotions, engage in problem-solving or avoid stressors^19^. Knowledge of their way of coping with stress is needed to increase the quality of treatment and to develop proper clinical interventions.

Although investigations on the mental health of patients with ichthyoses have led to advances in the field, earlier studies solely focused on clinical groups and lacked control groups with healthy participants to compare the results^20–22^. Other studies showed increased levels of anxiety in people living with ichthyoses^20^, but to our knowledge, there are no studies on the strategies patients use to handle stressful situations.

Our study aims to compare state and trait anxiety levels between healthy adults and patients with Mendelian Disorders of Cornification assessing the frequency of using task-oriented, emotion-oriented, and avoidance strategies, with particular attention to avoidance strategies, such as distraction and social diversion.

## Materials and methods

The study cohort consisted of patients diagnosed with Mendelian Disorders of cornification (MeDOC). receiving treatment at the Dermatology Outpatient Clinic and the Dermatology Clinic at University Clinical Hospital No. 1 in Lublin between 2021 and 2023. The control group was selected based on medical records and a dermatological examination, and consisted of healthy volunteers without comorbidities, recruited among people who came to the Dermatology Outpatient Clinic to evaluate single skin lesions. The control group was matched in age and sex to the patients with genodermatoses. All participants provided informed consent to join the study. The Bioethics Committee at the Medical University of Lublin approved the study (KE-0254/91/2021, KE-0254/87/03/2023). All experiments were performed in accordance with relevant guidelines and regulations, and in accordance with the Declaration of Helsinki.

The study was conducted using questionnaires and psychometric tools for measuring patients’ emotional state and personality traits. The State and Trait Anxiety Inventory (STAI) analyzed the level of state and trait anxiety. The Coping Inventory for Stressful Situations (CISS) assessed how patients cope with adversity. Additionally, participants filled in a questionnaire developed by the authors, concerning sociodemographic data and the description of the specific disease symptoms. All these tools allowed for a comprehensive psychosocial assessment of the study participants. The questionnaires were appropriately adapted, and the study was conducted in accordance with methodological guidelines.

### Detailed description of tools used in the study

STAI serves as a tool to assess anxiety in two dimensions: as a momentary emotional state and as a stable personality trait. It comprises 40 statements, categorized into two subscales: X1 and X2, each containing 20 multiple-choice questions. In subscale X1, the respondents assess the extent to which the statements reflect their current emotional state, while in subscale X2, they assess the frequency of specific feelings. The scores obtained from both X1 and X2 subscales of the STAI fall within a reliability range of 0.70 to 0.90. This indicates that the STAI is consistently reliable in measuring anxiety experiences.

The CISS questionnaire is used to study how participants handle challenging and stressful situations. It comprises 48 statements, and respondents use a five-point Likert scale to answer. The CISS scale evaluates three primary coping strategies: task-oriented, emotion-oriented, and avoidance-oriented. The last strategy includes two forms: distraction and social diversion^23,24^. Cronbach’s alpha coefficients for the various subscales range from 0.78 to 0.90, indicating satisfactory internal consistency.

The author’s questionnaire comprises two sections. The first section covers common sociodemographic details such as sex, age, education, and place of residence. The second section collects data on symptoms associated with genodermatosis, including pain, scale color, skin itching, peeling, redness, infiltration, and the extent of the lesions. The authors developed this questionnaire to address the absence of standardized survey tools designed to measure and quantify the symptoms related to these conditions.

### Statistical analysis

The collected data were analyzed with MedCalc (version 15.8 PL) and Statistica (version 13 PL) software. The normality of the distribution of continuous variables was assessed by the D`Agostino-Pearson test. As a result of the non-normal distribution of continuous variables: (i) subsequent stages of statistical analysis employed non-parametric tests, (ii) the median served as the measure of data concentration, (iii) the interquartile range (IQR) and minimum-maximum range were used to depict data dispersion. The Mann-Whitney U test was applied when comparing the distribution of a continuous variable between two independent groups. Box-and-whisker plots were used to visualize the statistically significant results of the comparisons mentioned above. Dichotomized and categorical variables were presented as absolute numbers and percentages. Comparisons of such variables between groups were conducted with two types of tests. The Fisher exact test was used if two groups and categories of a variable were compared. The *Chi*-square test with the Yates correction was employed for comparisons of more than two variable categories. Statistically significant results in these comparisons were visualized using bar charts. Finally, the odds ratio test was used to estimate the risk/chance of specific clinical events based on the occurrence of a given variant of the analyzed categorical variable. In all statistical tests, results with alpha error (*p*) values below 0.05 were interpreted as significant, while results falling within the range of 0.05–0.07 were considered as demonstrating a trend towards statistical significance.

## Results

### Demographic, environmental, and educational characteristics of the study and control groups

The study (*n* = 29) and control (*n* = 30) groups were balanced with regard to demographics. Women constituted 72.4% (21 patients) in the study group and 70% (21 healthy volunteers) in the control group (*p* = 0.9340), and there were no significant differences in age (medians of 36 years and 36.5 years, respectively; *p* = 0.8258) or place of residence (urban residents comprising 62.1% [18 patients] and 83.3% [25 healthy volunteers], respectively; *p* = 0.1226). Subjects with higher education predominated in the control group (22 healthy volunteers), while they made up a smaller proportion in the study group (14 patients) (73.3% and 48.3%, respectively; *p* = 0.0880).

### Clinical characteristics of the study group

The key clinical details of the study group are summarized in Table 1. Disease-causing variables were found in all patients subjected to molecular testing. The study group comprised 29 participants with 7 different types of MeDOC. Ichthyosis vulgaris (10 patients) and lamellar ichthyosis (9 patients) were observed in the majority of patients (34.5% and 31%, respectively). Five individuals were diagnosed with palmoplantar keratoderma; two had erythrodermic ichthyotis, while ichthyosis with confetti, Autosomal Recessive Congenital Ichthyosis (ARCI), and Erythrokeratodermia Variabilis (EV)-like skin lesions were reported in single patient each. We described the group of investigated patients in a previous paper^25^.

Table 1 presents the clinical characteristics of the studied patients, while Table 2 includes detailed characteristics. Sixteen patients (55.2%) presented with lesions on exposed body parts. Scales covering the entire body were present in 14 respondents (48.3%), with a median percentage of affected body surface area of 90% (min-max: 1–100%). In most cases, the scales were of different colors. The median and min-max scores for redness, peeling, itching, and pain, measured using the visual analogue scale (VAS) were as follows: 5 (1–10), 7 (1–10), 5 (1–10) and 2 (0–8). Twelve respondents presented with infiltration (41.4%) and 8 with ectropion (27.6%). Hearing loss, ear plugging, and eyelid closure were observed in 6, 13 and 17 patients, respectively (20.7%, 44.8% and 58.6%, respectively). Anhidrosis, hypohidrosis and hyperhidrosis were found in 6, 10, and 10 patients, respectively (10%, 33.3% and 33.3%, respectively). Ten patients (34.5%) suffered from sleep disorders. The median score on the Karnofsky scale used to assess functional impairment was 90 and the scores ranged from 25 to 100 points.

**Table 1:** Patient characteristics.

Patients characteristics		Study group [*n* = 29]
Sex	Men	8 (27.6%)
	Women	21 (72.4%)
Age [years]	Median [IQR] (Min-Max)	36 [33–45] (16–79)
Genodermatosis type	Autosomal Recessive Congenital Ichthyosis (ARCI)	1 (3.4%)
	Erythrokeratodermia variabilis (EV)	1 (3.4%)
	Erythrodermic ichthyotis	2 (6.9%)
	Ichthyosis with confetti	1 (3.4%)
	Lamellar ichthyosis	9 (31%)
	Keratoderma palmoplantar	5 (17.2%)
	Ichthyosis vulgaris	10 (34.5%)
Scales on exposed parts of the body	No	13 (44.8%)
	Yes	16 (55.2%)
% of body surface covered in lesions	Median [IQR] (Min-Max)	90 [57–100] (1-100)
The whole body is covered in scales	No	15 (51.7%)
	Yes	14 (48.3%)
Color of scales	Transparent	1 (3.4%)
	White	3 (10.3%)
	Yellow	4 (13.8%)
	Brown	5 (17.2%)
	Beige	6 (20.7%)
	Multicolored	10 (34.5%)
Itching	Median [IQR] (Min-Max)	5 [4–6] (1–10)
Redness	Median [IQR] (Min-Max)	5 [4–6] (1–10)
Exfoliation	Median [IQR] (Min-Max)	7 [5–8] (1–10)
Infiltration	No	17 (58.6%)
	Yes	12 (41.4%)
Pain (VAS)	Median [IQR] (Min-Max)	2 [1–4] (0–8)
Level of sweating	Absolutely no sweating	3 (10.3%)
	No sweating	10 (34.5%)
	Normal sweating	6 (20.7%)
	Increased sweating	10 (34.5%)
Ectropion	No	21 (72.4%)
	Yes	8 (27.6%)
Sleep disorders	No	19 (65.5%)
	Yes	10 (34.5%)
Inability to fully close the eyelids	No	12 (41.4%)
	Yes	17 (58.6%)
The feeling of ear plugging	No	16 (55.2%)
	Yes	13 (44.8%)
Hearing loss	No	23 (79.3%)
	Yes	6 (20.7%)
Performance status (Karnofsky scale)	Median [IQR] (Min-Max)	90 [70–96] (25–100)

**Table 2:**
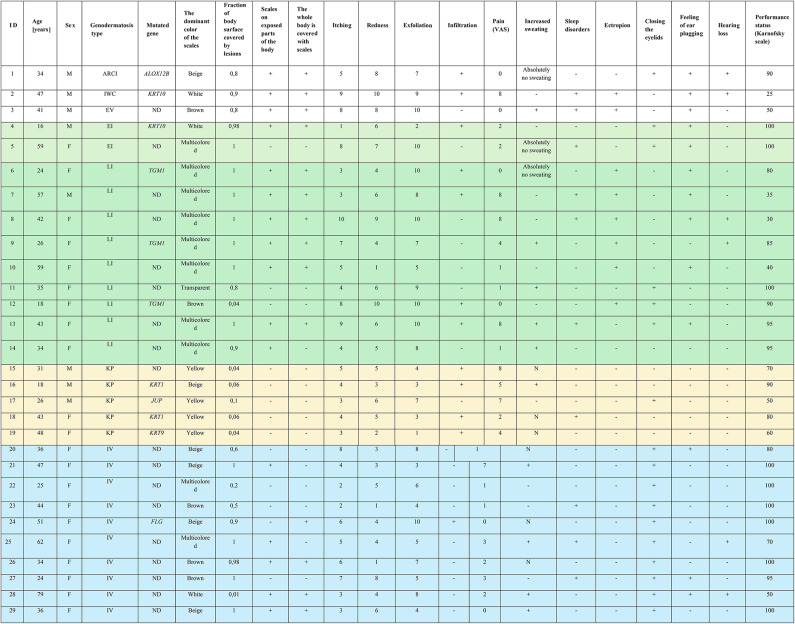
Detailed patients characteristics and their confirmed mutations.

*ND* no data, *ARCI* autosomal recessive congenital ichthyosis, *EI* erythrodermic ichthyosis, *EV* erythrokeratodermia variabilis-like, *I* ichthyosis, *LI* lamellar ichthyosis, *IWC* ichthyosis with confetti, *KP* non-epidermolytic palmoplantar keratoderma, *IV* ichthyosis vulgaris, *F* female, *M* male.

### Comparison of the study and control groups in terms of the level of perceived anxiety

Average or high levels of trait anxiety were significantly more common in the study group compared to the control group (25 vs. 18 cases; 86.2% vs. 60%, respectively; *p* = 0.0488). Therefore, it was estimated that the risk of average or high levels of trait anxiety was over 4 times higher in the study group than in the control group (OR = 4.2, 95% CI 1.1–15; *p* = 0.0293). In addition, high levels of trait anxiety were also significantly more common in the study group compared to the control group (8 vs. 1 case; 27.6% vs. 3.3%, respectively; *p* = 0.0090). In contrast, there were no statistically significant differences in the level of state anxiety between the study and control groups. Detailed data is presented in Table 3.

**Table 3:** Comparison of the study and control group in terms of the level of state and trait anxiety assessed by the STAI questionnaire.

Variable	Study group [*n* = 29]	Control group [*n* = 30]	*p*
Level of state anxiety			
Low	7 (24.1%)	7 (23.3%)	0.8154
Average or high	22 (75.9%)	23 (76.7%)	
Level of state anxiety			
Low	7 (24.1%)	7 (23.3%)	0.9950
Average	18 (62.1%)	19 (63.3%)	
High	4 (13.8%)	4 (13.3%)	
Level of trait anxiety			
Low	4 (13.8%)	12 (40%)	0.0488*
Average or high	25 (86.2%)	18 (60%)	
Level of trait anxiety			
Low	4 (13.8%)	12 (40%)	0.0090*
Average	17 (58.6%)	17 (56.7%)	
High	8 (27.6%)	1 (3.3%)	

*Statistically significant result.

### Comparison of the study and control groups in terms of coping with adversity

The high level of emotion-oriented coping occurred significantly more often in the study group than in the control group (8 vs. 1 case; 27.6% vs. 3.3%; *p* = 0.0259). Thus, it was estimated that the risk of high levels of emotion-oriented coping was 11 times higher in the study group than in the control group (OR = 11, 95% CI 1.3–95.2; *p* = 0.0288). A significantly higher prevalence of average or high levels of avoidant-distracted coping was observed in patients in the study group compared to controls (25 vs. 14 cases; 86.2% vs. 46.7%; *p* = 0.0034). Therefore, it was estimated that the risk of average or high levels of avoidant-distracted coping was approximately 7 times higher in the study group compared to controls (OR = 7.1 95% CI 2-25.6; *p* = 0.0025). In addition, there was a significantly higher prevalence of high levels of avoidant-distracted coping in patients in the study group compared to controls (10 vs. 6 cases; 34.5% vs. 20%; *p* = 0.0058). Details of the comparison between the study and control groups in terms of stress coping styles assessed by the CISS questionnaire are presented in Table 4.

**Table 4:** Comparison of the study and control groups in terms of coping with adversity assessed by the CISS questionnaire.

Variable	Study group [*n* = 29]	Control group [*n* = 30]	*p*
Task-oriented coping			
Low	8 (27.6%)	4 (13.3%)	0.1641
Average	8 (27.6%)	15 (50%)	
High	13 (44.8%)	11 (36.7%)	
Task-oriented coping			
Low	8 (27.6%)	4 (13.3%)	0.3001
Average or High	21 (72.4%)	26 (86.7%)	
Emotion-oriented coping			
Low	11 (37.9%)	12 (40%)	0.0261*
Average	10 (34.5%)	17 (56.7%)	
High	8 (27.6%)	1 (3.3%)	
Emotion-oriented coping			
Low	21 (72.4%)	29 (96.7%)	0.0259*
Average or high	8 (27.6%)	1 (3.3%)	
Avoidant coping			
Low	7 (24.1%)	11 (36.7%)	0.2601
Average	12 (41.4%)	14 (46.7%)	
High	10 (34.5%)	5 (16.7%)	
Avoidant coping			
Low	7 (24.1%)	11 (36.7%)	0.4460
Average or high	22 (75.9%)	19 (63.3%)	
Avoidant-distracted coping			
Low	4 (13.8%)	16 (53.3%)	0.0058*
Average	15 (51.7%)	8 (26.7%)	
High	10 (34.5%)	6 (20%)	
Avoidant-distracted coping			
Low			0.0034*
Average or High	4 (13.8%)	16 (53.3%)	
High	25 (86.2%)	14 (46.7%)	
Avoidant-social coping			
Low	8 (27.6%)	7 (23.3%)	0.4668
Average	10 (34.5%)	15 (50%)	
High	11 (37.9%)	8 (26.7%)	
Avoidant-social coping			
Low	8 (27.6%)	7 (23.3%)	0.9394
Average or high	21 (72.4%)	23 (76.7%)	

*Statistically significant result.

### Relationship of demographic, education, social and clinical factors to anxiety levels in the study group

None of the demographic, social, education, and clinical factors assessed were significantly correlated with the level of anxiety (assessed using the STAI questionnaire). Detailed data on the association of demographic, social, education-related, and clinical factors with trait anxiety levels (assessed using the STAI questionnaire) in the study group are presented in Table 5.

**Table 5:** The association of demographic, social, education-related, and clinical factors with trait anxiety levels (assessed using the STAI questionnaire) in the study group.

Variable	Trait anxiety level		*p*
	Low [*n* = 4]	Average or high [*n* = 25]	
Sex			
Women	2 (9.5%)	19 (90.5%)	0.6328
Men	2 (25%)	6 (75%)	
Age [years], median [IQR] (Min-Max)	38.5 [22-56.5]	36 [29.8–47]	0.8992
Place of residence			
City	2 (11.1%)	16 (88.9%)	0.9847
Village	2 (18.2%)	9 (81.8%)	
Education			
Primary, Vocational, Secondary	2 (13.3%)	13 (86.7%)	0.6423
Higher	2 (14.3%)	12 (85.7%)	
Scales on exposed parts of the body			
No	3 (23.1%)	10 (76.9%)	0.4440
Yes	1 (6.2%)	15 (93.7%)	
Itching, median [IQR] (Min-Max)	4.5 [3.5–5.5]	5 [3–8]	0.7254
Exfoliation, median [IQR] (Min-Max)	6 [4-8.5]	7 [4-9.3]	0.7250
Redness, median [IQR] (Min-Max)	4 [3.5-5]	5 [3.8–7.3]	0.3544
The whole body is covered in scales			
No	3 (20%)	12 (80%)	0.6423
Yes	1 (7.1%)	13 (92.9%)	
Infiltrations			
No	2 (11.8%)	15 (88.2%)	0.8653
Yes	2 (16.7%)	10 (83.3%)	
Color of scales			
Beige, yellow or brown	2 (9.1%)	20 (90.9%)	0.5012
White	2 (28.6%)	5 (71.4%)	
% of body surface covered in lesions, median [IQR] (Min-Max)	50% [8-95%]	90% [42.5-100%]	0.5797
Pain, median [IQR] (Min-Max)	4 [1.5-6]	2 [1-4.8]	0.6305
Increased sweating			
No	2 (10.6%)	17 (89.4%)	0.8093
Yes	2 (20%)	8 (80%)	
Ectropion			
No	4 (19%)	17 (81%)	0.4672
Yes	0 (0%)	8 (100%)	
Sleep disorders			
No	3 (15.8%)	16 (84.2%)	0.8912
Yes	1 (10%)	9 (90%)	
Inability to fully close the eyelids			
No	1 (8.3%)	11 (91.7%)	0.8653
Yes	3 (17.6%)	14 (82.4%)	
Feeling of ear plugging			
No	4 (25%)	12 (75%)	0.1614
Yes	0 (0%)	13 (100%)	
Hearing loss			
No	3 (13%)	20 (87%)	0.6632
Yes	1 (16.7%)	5 (83.3%)	
Performance status (Karnofsky scale) Median [IQR] (Min-Max)	80 [60–95]	90 [57.5–100]	0.8218

*Statistically significant result.

### Relationship of demographic, social, education, and clinical factors to stress coping mechanisms in the study group

#### Emotion-oriented coping

Patients with a high level of emotion-oriented coping had a significantly higher median redness score compared to those with a low or average level of emotion-oriented coping (7.5 vs. 5; *p* = 0.0385). None of the other demographic, social, education, and clinical factors assessed were significantly associated with this style of coping (assessed by the CISS questionnaire).

#### Avoidant-distracted coping

None of the demographic, social, education, and clinical factors assessed were significantly associated with avoidant-distracted coping (assessed according to the CISS questionnaire).

Detailed data on the association of demographic, social, education-related, and clinical factors with emotion-oriented coping and with avoidant-distracted coping (assessed according to the CISS questionnaire) in the study group are presented in Table 6.

**Table 6:** The association of demographic, social, education, and clinical factors with emotion-oriented coping and with avoidant-distracted coping (assessed according to the CISS questionnaire) in the study group.

Variable	Emotion-oriented coping		*p*	Avoidant-distracted coping		*p*
	Low or average [*n* = 21]	High [*n* = 8]		Low [*n* = 4]	Average or high [*n* = 25]	
Sex						
Women	15 (71.4%)	6 (28.6%)	0.7853	3 (14.3%)	18 (85.7%)	0.6328
Men	6 (75%)	2 (25%)		1 (12.5%)	7 (87.5%)	
Age [years], Median [IQR] (Min-Max)	36 [26-44.8]	45 [29–58]	0.3660	34.5 [21–51]	36 [29.8–47.3]	0.6806
Place of residence						
City	13 (72.2%)	5 (27.8%)	0.6902	4 (22.2%)	14 (77.8%)	0.2589
Village	8 (72.7%)	3 (27.3%)		0 (0%)	11 (100%)	
Education						
Primary, vocational, secondary	10 (66.7%)	5 (33.3%)	0.7634	3 (20%)	12 (80%)	0.6423
Higher	11 (78.6%)	3 (21.4%)		1 (7.1%)	13 (92.9%)	
Scales on exposed parts of the body						
No	9 (69.2%)	4 (30.8%)	0.9426	0 (0%)	13 (100%)	0.1614
Yes	12 (75%)	4 (25%)		4 (25%)	12 (75%)	
Itching, Median [IQR] (Min-Max)	4 [3-6.3]	6 [3–8]	0.4450	6 [3–8]	4 [3-7.3]	0.6780
Exfoliation, Median [IQR] (Min-Max)	7 [4–9]	8 [6–10]	0.3118	6 [3.5–8.5]	7 [4-9.3]	0.6315
Redness, Median [IQR] (Min-Max)	5 [3–6]	7.5 [5-8.5]	0.0385*	5 [2.5-6]	5 [3.8–7.3]	0.5655
The whole body is covered in scales						
No	11 (73.3%)	4 (26.7%)	0.7634	0 (0%)	15 (100%)	0.0909
Yes	10 (71.4%)	4 (28.6%)		4 (28.6%)	10 (71.4%)	
Infiltrations						
No	13 (76.5%)	4 (23.5%)	0.8729	2 (11.8%)	15 (88.2%)	0.8653
Yes	8 (66.7%)	4 (33.3%)		2 (16.7%)	10 (83.3%)	
Color of scales						
Beige, yellow or brown	15 (68.2%)	7 (31.8%)	0.6756	2 (9.1%)	20 (90.9%)	0.5012
White	6 (85.7%)	1 (14.3%)		2 (28.6%)	5 (71.4%)	
% of body surface covered in lesions, Median [IQR] (Min-Max)	90% [42.5-100%]	90% [4-100%]	0.8016	100% [99-100%]	80% [9-100%]	0.0546
Pain, Median [IQR] (Min-Max)	2 [1-5.5]	2.5 [1–6]	0.6207	3 [1.5-6]	2 [0.8–5.5]	0.4415
Increased sweating						
No	12 (63.2%)	7 (36.8%)	0.2713	2 (10.5%)	17 (89.5%)	0.8912
Yes	9 (90%)	1 (10%)		2 (20%)	8 (80%)	
Ectropion						
No	16 (76.2%)	5 (23.8%)	0.7853	2 (9.5%)	19 (90.5%)	0.6328
Yes	5 (62.5%)	3 (37.5%)		2 (25%)	6 (75%)	
Sleep disorders						
No	15 (78.9%)	4 (21.1%)	0.5170	3 (15.8%)	16 (84.2%)	0.8912
Yes	6 (60%)	4 (40%)		1 (10%)	9 (90%)	
Inability to fully close the eyelids						
No	9 (75%)	3 (25%)	0.8729	2 (16.7%)	10 (83.3%)	0.8653
Yes	12 (70.6%)	5 (29.4%)		2 (11.8%)	15 (88.2%)	
Feeling of ear plugging						
No	14 (87.5%)	2 (12.5%)	0.1099	1 (6.2%)	15 (93.7%)	0.4440
Yes	7 (53.8%)	6 (46.2%)		3 (23.1%)	10 (76.9%)	
Hearing loss						
No	18 (78.3%)	5 (21.7%)	0.3862	3 (13%)	20 (87%)	0.6632
Yes	3 (50%)	3 (50%)		1 (16.7%)	5 (83.3%)	
Performance status (Karnofsky scale), Median [IQR] (Min-Max)	90 [70–100]	75 [42.5–92.5]	0.2146	90 [62.5–97.5]	90 [57.5–100]	0.8976

*Statistically significant result.

## Discussion

Mendelian Disorders of cornification (MeDOC) affect multiple aspects of patients’ lives, including their professional, sexual, and social activities. Anxiety and depressive disorders may also develop due to biological and psychosocial factors associated with the disease^26^. In our study, we demonstrated that the majority of patients with MeDOC experience high levels of anxiety. Patients with MeDOC exhibit heightened reactivity to stimuli that others perceive as insignificant, driven by anxiety related to social rejection due to visible skin lesions. The perception of everyday stimuli as potentially threatening may trigger intense anxiety disproportionate to the actual risk^15,27^. Wciórka highlighted the psychological context of anxiety in various clinical syndromes, emphasizing that its manifestation depends on the presence of other symptoms^28^. Anxiety, depending on its nature, may be the predominant symptom of a disorder, the basis for a distinct diagnosis, or a secondary symptom. It may also develop secondarily in the course of conditions such as depression or schizophrenia^28^.

The literature on anxiety in skin diseases provides a wealth of relevant information on the impact of such conditions on patients’ mental health. A study by Cortés et al. of 24 patients with ichthyosis pallidus found varying levels of anxiety, with 8% experiencing minimal anxiety, 42% experiencing mild anxiety, 33% experiencing moderate anxiety, and 17% experiencing severe anxiety. Despite the use of a different scale, the results indicate higher levels of anxiety among people with ichthyosis compared to the control group, confirming the negative impact of this disease on mental health^29^. Anxiety significantly affects daily life, as confirmed by a study by Sun et al. analyzing the relationship between quality of life and anxiety in people with ichthyosis. The results of their study, despite methodological differences, show a positive correlation between anxiety and the Dermatology Life Quality Index (DLQI) (0.36–0.65), meaning that higher levels of anxiety are associated with a reduced quality of life. They also found that anxiety is a significant predictor of a reduced quality of life in areas such as leisure (OR = 1.08), and work and school (OR = 1.11). Adults with anxiety are more likely to have a decreased quality of life. These findings support our study, highlighting that anxiety significantly reduces quality of life and affects overall well-being^20^.

Acceptance of the disease is also a key factor in mitigating its psychological impact. Patients who accept their skin condition are less likely to experience heightened anxiety, obsessive-compulsive symptoms, or somatic complaints^30^. A lack of acceptance may lead to increased anxiety and diminished well-being. Severe depressive and anxiety symptoms may also occur in certain patient groups, such as women, possibly due to difficulties in accepting the disease and its impact on self-esteem. This may contribute to feelings of social exclusion and increased exposure to stress. Additionally, individuals with difficulties in recognizing and describing emotions (alexithymia) are more likely to experience increased somatization and anxiety, in line with the psychosomatic theory, which suggests that emotions manifest themselves through somatic symptoms^31–34^.

Our study highlights the importance of stress-coping strategies in individuals with MeDOC, including ichthyosis. We aimed to investigate the mechanisms patients use to manage situations that disrupt their emotional balance, particularly those that are challenging and potentially distressing. The results demonstrated that individuals with MeDOC are significantly more likely to employ emotion-focused coping strategies and avoidance compared to the control group. These findings suggest that individuals with MeDOC often focus on their own emotions and the disease itself, which may be part of an adaptive process—allowing them to regulate their emotional responses. At the same time, a tendency to avoid difficult situations may serve as a coping mechanism to manage stress by distancing themselves from the problem. In the context of social interactions, the avoidance of specific situations may stem from a fear of negative evaluation by others, although our study did not directly assess self-stigmatization. Nevertheless, a tendency toward avoidance strategies may limit patients’ social engagement and hinder their full adaptation to life with the disease^35^. Studies show that patients with skin diseases use a variety of coping strategies, often combining several methods. Most often, they hide skin lesions, educate those around them, correct misconceptions about the contagiousness of the disease, or convince themselves that others will not understand them. Some patients note that the reactions of others may be due to insensitivity or rudeness. These mechanisms are designed to avoid negative social reactions, but do not always improve quality of life. Concealing skin lesions and avoiding social interactions can exacerbate isolation, while correcting misconceptions can reduce stigma and improve social relationships and quality of life^36–39^. A study by Slomian and colleagues found that patients with psoriasis had higher levels of anxiety than healthy individuals, were more likely to use avoidance and emotional strategies, and were less likely to use task-based strategies. However, these strategies had no significant effect on anxiety levels^40^.

Other studies have revealed sex differences in adaptive strategies: women are more likely to use emotional support and religion, while men prefer distraction and emotional expression. The results suggest that treatment of chronic dermatological diseases should be tailored to the individual patient’s needs and life context. The personalization of coping strategies can improve quality of life and reduce the negative impact of the disease on mental health^17,36,38,39^.

Our study underscores the importance of psychosocial support for people with skin diseases and the need for a holistic approach in treatment. Doctors should pay attention to patients’ mental state, regularly asking about their well-being and not underestimating emotional problems. It is also important to refer patients to professional psychological therapy, which can significantly improve patients’ ability to cope with the disease and manage its impact on their daily lives. Integrating medical and psychological support is crucial to fully and effectively support patients. Physicians need to be aware that the psychological aspects of skin diseases are as important as the physical ones and can significantly affect quality of life. Regular monitoring of emotional status and appropriate psychological support can help alleviate the stress and anxiety that often accompany chronic skin diseases, which can ultimately improve patients’ overall health.

## Conclusions

Patients with MeDOC are at a higher risk of anxiety, often exhibiting elevated anxiety levels regardless of demographic or clinical factors. They predominantly use emotion-focused and avoidant-distracted coping strategies, with the former linked to skin redness. Despite these psychological differences, their personality traits remain comparable to those of healthy individuals. These findings highlight the need for psychological support tailored to their coping mechanisms. Psychological support should be an integral part of the treatment process for individuals with genodermatoses. Combining psychological interventions with standard dermatological care can significantly improve patients’ quality of life. Regular psychological consultations should be a key element of care for individuals with MeDOC. Monitoring anxiety and depression, along with assessing coping strategies, facilitates the early identification of those in need of additional support and ensures timely intervention. An interdisciplinary approach that integrates dermatological and psychological care can greatly enhance patients’ well-being. Education, group support, and personalized therapeutic strategies play a crucial role in helping individuals with MeDOC navigate daily challenges associated with their condition.

## Data Availability

The data that support the findings of this study are available from the corresponding author, upon reasonable request.
